# Spigelian hernia in gynaecology

**DOI:** 10.1186/s10397-017-1010-8

**Published:** 2017-05-15

**Authors:** Anastasia Ussia, Fabio Imperato, Larissa Schindler, Arnaud Wattiez, Philippe R. Koninckx

**Affiliations:** 1 Villa Del Rosario, Rome, Italy; 2 Gemelli Hospitals, Università Cattolica, Rome, Italy; 3 Latifa Hospital, Dubai, United Arab Emirates; 4 Department of Obstetrics and Gynecology, Catholic University Leuven, University Hospital, Gasthuisberg, B-3000 Leuven, Belgium. Vuilenbos 2 3360, Bierbeek, Belgium

**Keywords:** Endometriosis, Spigelian hernia, Pelvic pain, Deep endometriosis

## Abstract

**Background:**

A Spigelian hernia is a rare hernia through the Spigelian fascia between the rectus muscle and the semilunar line. This hernia is well known in surgery. Symptoms vary from insidious to localised pain, an intermittent mass and/or a bowel obstruction.

**Results:**

The Spigelian hernia is poorly known in gynaecology. Spigelian hernias may be causally related to secondary trocar insertion. This review is written to increase awareness in gynaecology and is illustrated by a case report in which the diagnosis was missed for 4 years even by laparoscopy. Smaller hernias risk not to be diagnosed and will thus not be treated. Even larger Spigelian hernias might not be recognised and treated appropriately.

**Conclusions:**

The gynaecologist should consider a Spigelian hernia in women with localised pain in the abdominal wall lateral of the rectus muscle some 5 cm below the umbilicus. Smaller hernias can be closed by laparoscopy without a mesh. Larger hernias require a mesh repair.

## Background

Spigelian hernia or Spiegel hernia was recognised by Josef T. Klinkosh in 1764, and named after Adriaan van der Spieghel, a Flemish anatomist who described the semilunar line in 1645 [1]. The Spigelian fascia is the aponeurotic layer between the rectus abdominis muscle medially and the semilunar line laterally. This hernia can present as a localised pain, as an intermittent mass or as a bowel obstruction [2]. Symptoms however can also be insidious and non-specific. Spigelian hernia carries a risk of bowel incarceration and should therefore be repaired [3]. For smaller Spigelian hernias, a mesh-free laparoscopic suture repair is feasible [4, 5].

Spigelian hernia is rare, representing 1 to 2% of all abdominal hernias. It occurs mostly in women over 60 years of age [6] but can occur at a younger age [7], even in neonates [8, 9]. The aetiology is unclear; obesity with rapid weight loss, multiple pregnancies and chronic obstructive pulmonary disease (COPD) are considered predisposing factors. Recently, it was suggested that Spigelian hernia could be caused by a previous trocar insertion [10–13].

A missed diagnosis for 4 years in a woman operated 4 years previously for (deep) endometriosis, and the potential causal relationship with trocar insertion prompted us to review the literature.

## Methods

### Literature review

PubMed was screened for ‘Spigelian hernia’ generating 486 articles of which 126 since 1 January 2010, of which only three in a gynaecological journal [10, 13]. Most articles are case reports—the first publication in gynaecological literature was in 1992 [14]—or small series, the largest describing 40 cases [4]. Considering that five reviews were published since 11 January 2015 [15–20] and that articles before 1 January 2010 were almost exclusively case reports adding little to ethology, diagnosis or treatment, the review was limited to the last 6 years.

## Results

### Symptoms of a Spigelian hernia

Spigelian hernias can present as acute small bowel obstructions [15, 21] even of the colon [22]. Most Spigelian hernias present a localised hernia, or as local pain with or without a hernia sac [15–20]. Symptoms however can also be insidious and vague. A Spigelian hernia can harbour an endometrioma [23], even an acute appendicitis [8, 24] or an incarceration of omentum [25].

### Diagnosis of a Spigelian hernia

According to the surgical literature, dealing with larger and symptomatic hernias, the diagnosis is based on clinical examination helped by ultrasound or computed tomography [16]. Since most hernias reduce spontaneously in a supine position, the diagnosis, especially of smaller hernias, remains difficult and ultrasound diagnosis has been suggested to be performed in a standing position [8]. Diagnosis remains a diagnostic challenge [26] and requires a high index of suspicion given the lack of consistent symptoms and signs [27]. Larger or symptomatic Spigelian hernia’s can be suspected by CAT scan [28].

### Treatment of a Spigelian hernia

The laparoscopic repair is effective but used in few reference centres. A recent systematic review of the laparoscopic repair of a Spigelian hernia [15] identified 55 articles and concluded that laparoscopic repair of the Spigelian hernia is a safe and acceptable method. Although intraperitoneal mesh technique is the most popular repair method, various techniques were used in these 237 repairs without a single randomised trial.

An extra peritoneal mesh repair remains the gold standard [16]. Also during these repairs, laparoscopy is a useful aid as discussed in two recent case reports [29, 30].

Recurrences are around 3%. No predictive factors were identified [6].

## Case report

Our patient, who was 35 years of age, underwent laparoscopic surgery for excision of superficial/deep/ovarian endometriosis. A week after laparoscopy she suddenly developed pain in the right fossa which severely limited physical activity. She had difficulty to elevate the right leg, difficulty to walk, and felt severe pain when getting up from a chair by a rotating movement of the body. Intercourse was reported to be almost impossible because of this pain. In the absence of a positive finding during clinical exams, ultrasound, MRI and CAT scans, a tentative non-specific diagnosis was made that a nerve lesion must have occurred during the previous surgery either the genito-femoralis or the pudendal nerve. The local pain in the wall was considered a nerve entrapment or pain in the scar of the trocar, and local infiltrations with cortisone were made. Because of persisting severe complaints in the absence of a clinical finding, she was subsequently thought to exaggerate her symptoms and anti-depressive drugs were given. Although a triathlon runner before, she could not do any exercise and gained more than 20 kg. Two years later, a second laparoscopy was performed to exclude progression of the endometriosis but only a few spots of superficial endometriosis were reported. She then conceived by IVF (3 cycles), and during the Caesarean section, ‘pelvic endometriosis’ and adherences were reported but not treated. Subsequently, medical therapy with oral contraception was started. For the local pain, repetitive infiltrations in the wall were given, without a positive result.

When the patient visited us, 4 years after the first laparoscopy, she still had severe pain with difficulty to walk, to do physical activity, to have intercourse and to get up from a chair. Other symptoms were vague and not specific. Clinical exam revealed a severe localised pain in the right fossa upon palpation also with contracted abdominal muscles. The gynaecological exam and a transvaginal ultrasound and a MRI were normal, except some vague pain in the right vaginal fornix on deep palpation, some tenderness over the right Alcock canal and a small cystic ovarian endometriosis in both ovaries. Since the patient insisted, we accepted to perform a (third) diagnostic laparoscopy and we hoped to find a cause and solution for this persisting severe pain preventing physical activity. The cystic ovarian endometriosis was not considered sufficient to explain the pain. Also an Alcock syndrome, persistent deep endometriosis with perineal pain, or pain from the appendix seemed remote possibilities.

**Fig. 1:**
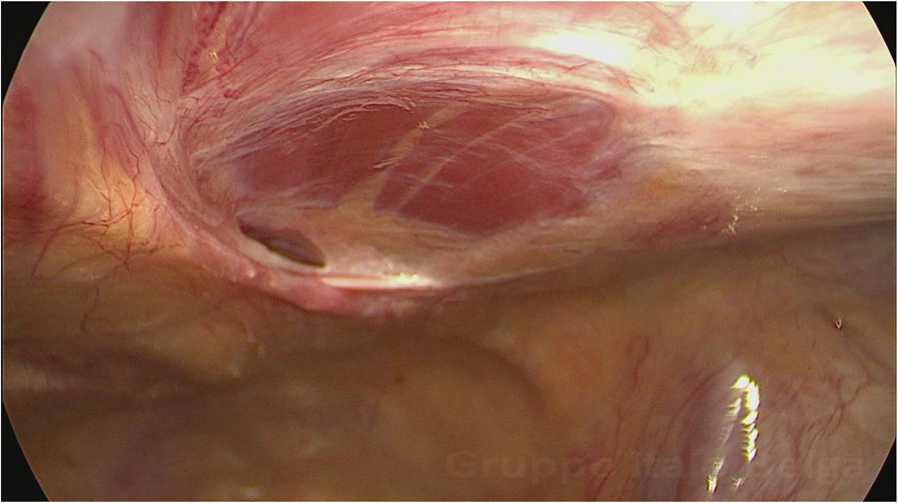
Spigelian hernia under rectus muscle causing severe pain limiting physical activity

The main finding during laparoscopy was a Spigelian hernia under the rectus muscle (Fig. 1) exactly where the pain was found by abdominal palpation. This hernia was excised and repaired with interrupted stitches of vicryl 0. Two small ovarian endometriosis cysts of less than 1 cm in diameter were excised from the left and right ovaries. A superficial plaque of endometriosis overlying the right ischial spine up to the bowel was excised. Because of the suspicion of Alcock syndrome, a dissection of the lateral side wall was performed exposing the right ischial spine, but no endometriosis was found. Because of the symptoms and despite having a normal appearance, an appendectomy was performed. To our surprise, the morning after surgery, the patient reported to be pain free and she was able to walk normally. A few days later she resumed normal physical activity, something that had not been possible for over 4 years.

The pathology of the hernia wall and of the appendix was reported as hernia lipoma and chronic appendicitis respectively. Pathology also confirmed the superficial pelvic and the cystic ovarian endometriosis.

## Discussion

Although we did not recognise this hernia as a Spigelian hernia, a hernia repair was performed since AU had seen many years before a similar hernia when assisting her brother who is a general surgeon. We were surprised and confused by the discrepancy between the severe pain symptoms, the minor surgery performed for endometriosis and the appendectomy and the immediate pain relief after surgery. Weeks later, after discussing this observation with other endoscopists, we realised that the hernia had been a Spigelian hernia. Since we consider that the endometriosis surgery performed and the appendectomy could not explain the immediate and complete pain relief within 24 h, we suspect that the Spigelian hernia had been the cause of the pain. This prompted us to review the literature to find that awareness of Spigelian hernia among gynaecological surgeons seems limited and that it might be caused by trocar insertion. Having been misled by the symptoms, we considered that reporting our lack of knowledge could increase awareness of Spigelian hernia.

Spigelian hernias generally present as an acute abdomen secondary to bowel strangulation or as a mass protruding from the wall. The prevalence and symptoms of smaller Spigelian hernias are unclear, since they risk not to be diagnosed, at least not by gynaecological laparoscopists. We indeed do not recognise what we do not know. Moreover, in gynaecology a localised hypogastric pain lateral to the rectus muscle which can be reproduced during contraction of the rectus muscle risks to be diagnosed as a nerve entrapment—as we did—and thus treated by local infiltration—as was done repetitively in this patient. An endometriosis lesion in the abdominal wall can be considered especially when the pain is cyclical and in the absence of a palpable lesion treatment will be conservative. Anyway, most of these women will not undergo a laparoscopy and the diagnosis risks not to be made.

A Spiegel hernia with such severe debilitating pain limiting physical activity is rare. The severe pain with difficulty to walk, work, move the right leg and have intercourse were erroneously considered for 4 years as the consequence of a nerve trauma while the local pain was considered a nerve entrapment in the wall. Only afterwards after making the diagnosis, we realised that this had been an antalgic reaction. Illustrative was the comment of the husband the morning of the day after surgery: ‘The problem is solved: my wife gets up straight from the bed whereas before she made a rotating movement.’ It also illustrates how confusing the history of a previous deep endometriosis surgery can be. Indeed an extensive dissection for deep endometriosis with afterwards still pain and difficulties to elevate or cross the right leg and to walk, raises suspicion of incomplete surgery and/or pelvic nerve damage. For this, repeat surgery will rarely be proposed and medical treatment will be given. Also in this case, we would not have performed a laparoscopy and thus would have missed the diagnosis without the insistent request of the patient.

The Spigelian hernia is suggested to be the cause of the pain since the pain symptoms, both the spontaneous pain and the pain during palpation of the wall had disappeared immediately after surgery. The small endometriotic cysts in the ovaries, and the superficial endometriosis were not painful during clinical exam before surgery and are unlikely to have caused the severe pain symptoms limiting physical activity nor the local pain in the wall. This also applies to the appendix. The fibrosis around the ischial spine may have given some Alcock-like symptoms, but does not explain the pain symptoms. It remains unclear why this Spigelian hernia caused such severe pain, since no bowel or omentum entrapment, or local inflammation was found.

Important is that a Spigelian hernia might be caused by a previous trocar insertion. This was suggested [10] and also in this woman the pain started acutely 1 week after the first surgery. It would therefore not be surprising that (smaller) Spigelian hernias are much more frequent than suspected since in the absence of severe symptoms they risk to remain undiagnosed. We gynaecological endoscopists should consider a Spigelian hernia in women with localised pain lateral to the rectus muscle 5 cm below the umbilicus mimicking a nerve entrapment especially after previous laparoscopy. The causal relationship with trocar insertion might be an argument to use secondary trocars with an atraumatic round instead of a triangular tip in order to decrease the muscular and fascial trauma [31]. The suggestion to use round tip trocars can moreover be generalised since incisional hernias also occur after 5-mm trocar insertion especially following extensive manipulation [32].

The specific location of the Spigelian hernia moreover suggests that it might be wise to avoid the angle between the semilunar line and the rectus abdominis muscle when inserting a secondary trocar. In a gynaecological surgery, a larger 10-mm secondary trocar is generally used on the left side. Most Spigelian hernias occur on the right side possibly related to appendectomies. The number of recent publications indeed suggests an increase in the occurrence;

## Conclusions

In conclusion, this case report intends to increase awareness among gynaecological laparoscopists of Spigelian hernia. It is suggested that previous trocar insertion might be causally related. It is speculated that the prevalence of smaller Spigelian hernias could be higher than believed today. It is suggested that it might be wise to perform a diagnostic laparoscopy in women with spontaneous pain and pain on palpation, in the angle between the rectus abdominis muscle and the semilunar line, especially after a previous laparoscopy or with an acute onset of pain thereafter. This case report also illustrates how misleading deep endometriosis can be. It also is a lesson in humility. Indeed we must admit that if in this woman a deep endometriosis nodule would have been found during surgery, compatible with her complaints, we might not have treated this Spigelian hernia since it was unknown to us at that moment.
